# Technological Innovations for Polymer Circularity: Upcycling, Biodegradation, and Processing

**DOI:** 10.3390/polym18202509

**Published:** 2026-10-14

**Authors:** Francesca Lionetto, Carlos Espinoza-González

**Affiliations:** 1 Department of Engineering for Innovation, University of Salento, Via Arnesano, 73100 Lecce, Italy; 2 Department of Advanced Materials, Center for Research in Applied Chemistry (CIQA), Blvd. Enrique Reyna 140, Saltillo 25294, Coahuila, Mexico

While polymer-based plastics play a vital role in key sectors such as packaging, construction and transportation, their material value is typically lost after a single use. Concerns regarding their accumulation in the environment were first evidenced in a 1972 report, which found plastic particles in the Sargasso Sea [1]. In a 2023 assessment, the United Nations Environment Programme (UNEP) reported that cumulative global plastic production had reached approximately 10 billion tons, of which, over 7 billion tons have already become waste, with less than 10% recycled and more than 75% discarded in landfills or natural ecosystems [2]. To address this growing waste crisis, the scientific community has significantly intensified its focus on sustainable material life cycles. Figure 1 illustrates the volume of research on polymer circularity, showing that scientific interest in this long-standing problem is increasing.

Two related streams of research have been motivated by these pressures:

1. How feedstocks, formulations, processing, and recovery can maintain or generate material value;
2. Whether these methods preserve performance during use and deliver better end-of-life outcomes.

While the two approaches differ in how they develop and evaluate circular pathways, research studies can address both.

In the first case, renewable oils can be used to produce polyols for polymer synthesis [3], and biomass residues can be turned into functional fillers [4]. The fact that a product is bio-based does not ensure that it will be biodegradable or have reduced life-cycle emissions; the results are determined by the type of feedstock, the process used, and the eventual fate of the product [5]. Waste valorization also involves the use of inorganic geopolymers obtained from industrial residues. Different recovery methods vary in what they can preserve: mechanical recycling preserves the polymer chains, whereas chemical or enzymatic processes are capable of recovering the building blocks for new polymers. The viability of these methods depends on the waste composition, pretreatment, material quality, and processing requirements [6,7].

The second stream involves materials after manufacturing. Heat, oxygen, and repeated processing can change the mechanical properties and the quality of the recycled [8]. Breaking materials down into smaller particles does not amount to complete biodegradation, and the rate varies with environmental conditions [9]. The results regarding ecotoxicity also depend on the properties of the particles and on the exposure: recent findings highlighted how crystallization kinetics affect the fluorescence behavior and ecotoxicological relevance of label-free PET nanoplastics in freshwater zooplankton [10]; while a meta-analysis found adverse effects on *Daphnia* reproduction (a genus of small freshwater crustaceans commonly called water fleas). Conversely, an outdoor study revealed no community-level effects from the tested polyester fibers at concentrations that are relevant to the environment [11,12]. When comparing pathways, a life-cycle assessment accounts for energy use, processing inputs, recovery yields, and the amount of primary materials displaced [13].

Figure 2a,b show the growth in the literature within these overlapping areas, supporting the broader trend illustrated in Figure 1. Because publications can relate to more than one topic, the numbers in the panels cannot be added together; Figure 1 provides the total number of distinct publications. Overall, these developments show that more research is being conducted to create circular pathways and to gather evidence to assess. However, growing publication activity does not, by itself, address the ongoing accumulation of plastic waste. What is urgently needed is to link advances in feedstocks, processing, and recovery with reliable evidence. This Special Issue “Technological Innovations for Polymer Circularity: Upcycling, Biodegradation, and Processing” addresses various aspects of this task. In the Contributions section, the original research articles are organized into two complementary streams:

1. Bio-based synthesis, biopolymers, and advanced recycling;
2. Durability, ecotoxicity, life-cycle assessment, and industrial waste valorization.

**Figure 2:**
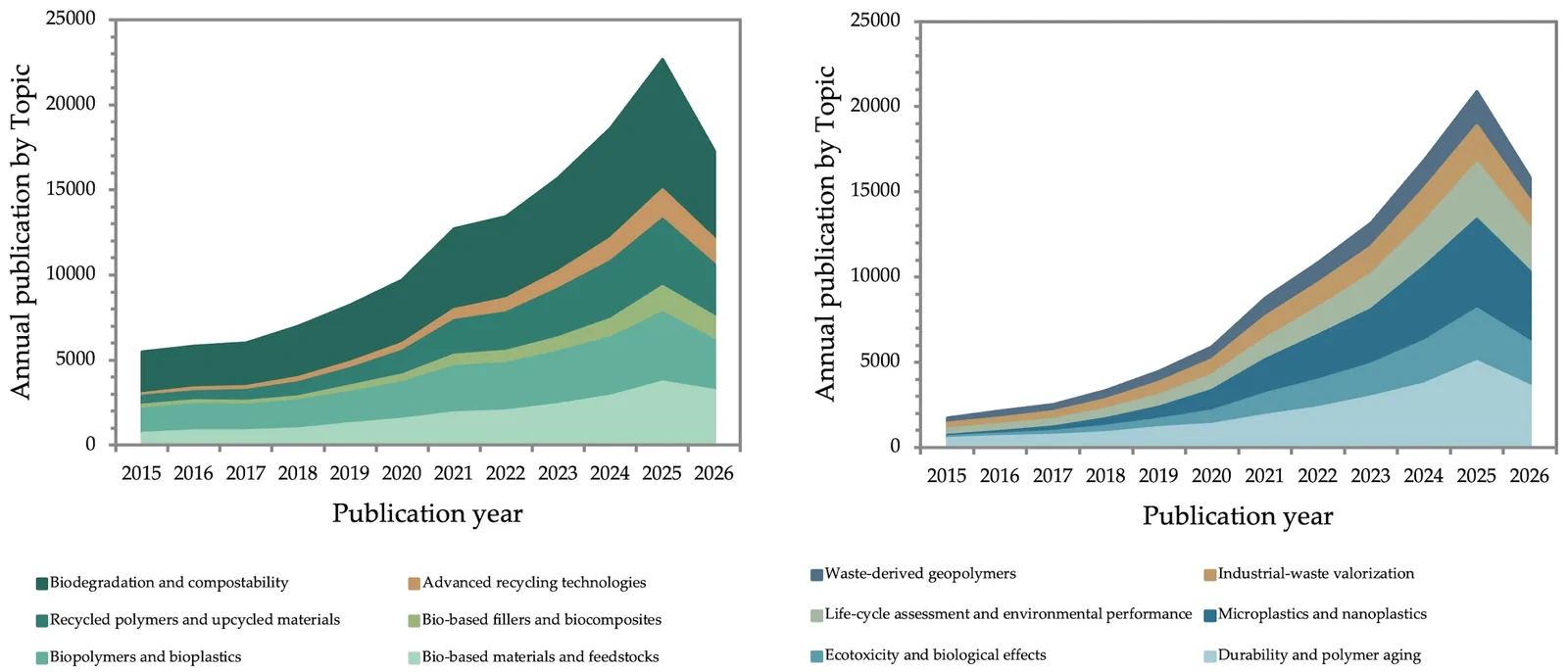
Annual publication trends across two streams of polymer circularity research. Panel (**a**) shows bio-based synthesis, biopolymers, and advanced recycling topics. Panel (**b**) shows durability, environmental effects, and waste valorization topics. Annual counts for 2015–2026 were obtained by applying topic-specific filters to the master search in the Web of Science database (Figure 1). Because publications may match multiple topics within or across panels, each stacked height represent topic matches rather than distinct publications. (*) Data for 2026 are incomplete, as the year is still in progress.

Then, we examine the methods and findings of these articles in further detail.

## 1. Contributions

### 1.1. Bio-Based Synthesis, Biopolymers, and Advanced Recycling

How can renewable resources and discarded polymers be turned into useful materials or recovered chemical components? The four contributions to this Special Issue in this research stream address this question at different stages of a material’s life cycle. They examine the synthesis of a bio-based building block, the use of biomass waste in a polymer composite, the conversion of discarded polymer into a new product, and the depolymerization of post-consumer waste. Together, these studies show that while a material’s source is only one aspect of circularity, the processes involved and the final products must also meet measurable criteria.

In the synthesis phase, Yang et al. address the limited exploration of amine-mediated ring opening of epoxidized soybean oil and the long reaction times associated with batch processing [14]. They carry out the reaction using cyclohexylamine in a continuous microfluidic reactor and investigate the effects of the temperature, residence time, catalyst loading, flow rate, and channel diameter on the product. Under the chosen conditions, the reaction achieves a hydroxyl value of 259.3 mg KOH/g in 60 min, compared with 155.1 mg KOH/g after 12 h in the corresponding batch experiment. This faster conversion is also accompanied by a narrower molecular-weight distribution and less evidence of etherification. The significance of their work is how it enables a renewable feedstock to be transformed into a more uniform chemical intermediate.

Another approach starts with a biomass residue, shifting attention from chemical synthesis to composite performance. Joe et al. examined biochar derived from banana and orange peels for use as a filler in PLA formulations intended for food packaging [15]. In formulations containing 1 to 3 wt.% biochar, the tensile strength, stiffness, and glass transition temperature all increased. This gains, however, come with trade-offs: the elongation at break decreases, and the maximum thermal degradation temperature moves downwards. The study shows how fruit peel waste can alter PLA properties, while also making clear that the suitability of the resulting formulations depends on the properties required for a particular application. Whether these formulations have suitable barrier properties or how readily they will biodegrade under use or disposal conditions have not yet been determined.

The focus then shifts from incorporating recovered biomass to maintaining the value of a polymer that has already been made. In this respect, Liu et al. use recycled poly(glycolic acid) (PGA) to produce electrospun nanofibers, which they then twist into yarns [16]. Since recycled PGA may have a lower molecular weight, they use graphene oxide (GO) and carbon nanotubes as reinforcing agents. Yarns containing 3 wt.% GO achieve a tensile strength of 21.36 MPa and an elastic modulus of 259.51 MPa; higher amounts of GO reduce the mechanical advantages, and various fillers also retard hydrolysis in water at 60 °C. In this recycling process, the polymer backbone is retained, and the material is given a new form, with its strength and degradation characteristics linked by the formulation.

Kumar et al. take a different route, recovering the chemical components of PET [17]. After examining three enzymes that degrade PET, they use crude LCCICCG produced in *E. coli* to depolymerize post-consumer waste without first purifying the enzyme. At a PET loading of 100 g/L, depolymerization reaches 78% for one waste stream and 50% for another after 60 h. The lower yield is associated with higher PET crystallinity, showing both the potential of the process using the crude enzyme and a limitation caused by the waste itself.

These studies provide complementary methods for creating or recovering value by improving bio-based synthesis, altering a biopolymer using biomass waste, producing a new product from recycled polymer, and recovering the components from discarded PET. Because they yield different results, each approach must be evaluated on its own merits. Factors such as conversion, material properties, degradation behavior, and feedstock variability should be considered, as the goal of polymer circularity shifts from a common objective to a series of specific experimental choices.

### 1.2. Durability, Ecotoxicity, Life-Cycle Assessment, and Industrial Waste Valorization

Complementing the first stream, evaluating the long-term behavior and broader sustainability of circular polymer pathways requires addressing material durability during service, environmental ecotoxicity, life-cycle impacts, and the valorization of industrial residues. At the material level, Li et al. [18] systematically investigated the thermo-oxidative aging mechanisms of ethylene–propylene polyolefins under accelerated thermal exposure (115–135 °C for up to 98 days). By tracking the mechanical performance, functional group evolution, thermal transitions, and molecular weight distribution, they identified a three-stage degradation process. In Stage I, thermal mobility induced secondary crystallization; in Stage II, progressive antioxidant consumption and accumulation of oxygenated species triggered a decline in crystallinity, structural voiding, and initial surface yellowing. In Stage III, complete antioxidant consumption accelerated β-scission leading to main-chain scission and loss of mechanical integrity.

Extending the evaluation from material-level degradation to system-level environmental performance, Walichnowska et al. [19] conducted a gate-to-gate life-cycle assessment (LCA) using the ReCiPe 2016 method on a mass bottle-packaging process using heat-shrinkable polyethylene film. They compared recycling with landfilling and, separately, examined electricity sources. Their findings demonstrated that mechanical recycling reduces the potential environmental impacts on human health and resource use compared to landfilling within the studied system boundaries, while switching from the Polish electricity mix to wind power reduces the potential damage to human health, ecosystems, and resources by approximately 91%, 89%, and 92%, respectively.

Extending this concern for the environmental impacts to industrial waste valorization, Terrones-Saeta et al. [20] developed waste-derived inorganic geopolymers by alkaline activation of lead mining residues with 5 wt.% NaOH solution to encapsulate up to 20 wt.% hazardous fly ash from municipal solid waste incineration. Toxicity characteristic leaching procedure (TCLP) tests confirmed that heavy metals (Pb, Cr, As, Cd, Ba) were released at concentrations below the regulatory limits under the tested conditions, while mechanical tests supported selected non-structural construction applications despite reduced strength with increasing ash content.

Finally, addressing the aquatic effects of polymer degradation products, Rajtar et al. [21] generated irregularly shaped PET micro/nanoplastics via two-stage ball milling in water to mimic natural weathering and assessed their ecotoxicity on freshwater zooplankton (*Daphnia magna* and *Thamnocephalus platyurus*). Although fluorescence microscopy confirmed rapid particle ingestion and accumulation in the digestive tract, acute exposure for 24–48 h across environmentally relevant and extreme concentrations (up to 1000 mg/L) revealed no acute mortality or swimming inhibition, indicating low short-term lethality under the tested conditions; longer-term effects remain to be assessed. Together, these four studies demonstrate how combining molecular durability mechanisms, quantitative life-cycle assessment, waste-derived material synthesis, and ecotoxicological screening provides a comprehensive framework to validate polymer circularity technologies.

## 2. Conclusions and Future Outlook

This Special Issue “Technological Innovations for Polymer Circularity: Upcycling, Biodegradation, and Processing” demonstrates that achieving true polymer circularity requires an integrated multi-scale strategy. Moving beyond linear plastic models demands aligning innovative chemical synthesis, bio-based biocomposites, process intensification, and enzymatic recycling with rigorous evaluations of material durability, ecotoxicological effects, and quantitative life-cycle impacts. By organizing the contributions into two complementary streams, one focused on bio-based materials and advanced recovery and the other on the long-term durability, environmental safety, and waste valorization, this collection highlights that material-level performance and system-level sustainability are deeply interconnected. As shown in the featured studies, understanding thermo-oxidative degradation mechanisms, encapsulating hazardous industrial residues within inorganic matrices, and screening micro/nanoplastics under realistic exposure conditions provide essential criteria for validating sustainable end-of-life pathways. Ultimately, closing the loop in the polymer economy relies on sustained interdisciplinary collaboration across polymer chemistry, materials engineering, ecotoxicology, and industrial ecology to deliver high-performing materials with demonstrable circularity benefits. We express our sincere gratitude to all the authors for contributing their cutting-edge research, to the peer reviewers for their dedicated evaluations, and to the editorial team of *Polymers* for their continuous support in bringing this Special Issue to fruition.

## Figures and Tables

**Figure 1:**
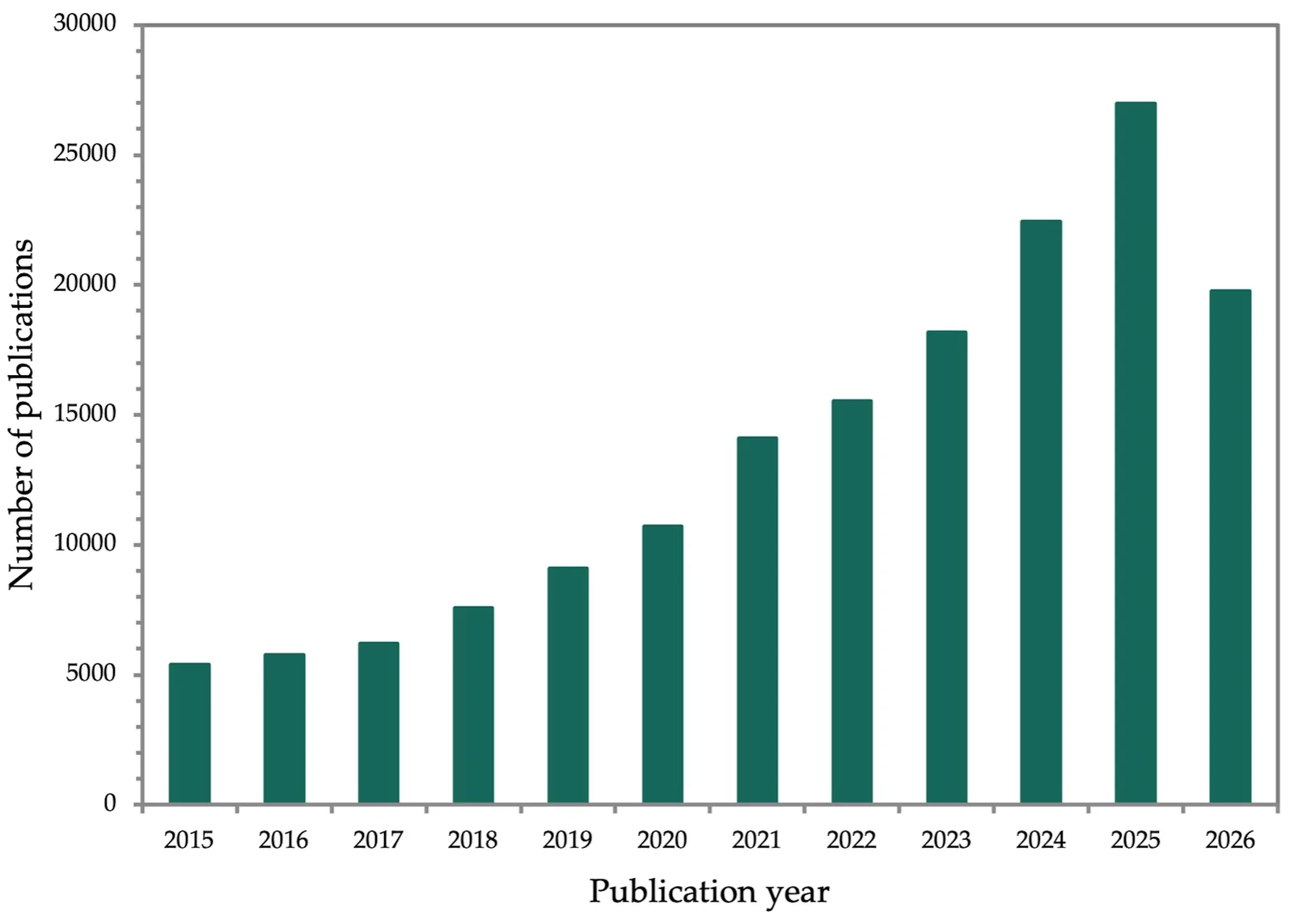
Annual publications on polymer circularity. Records from 2015 to 2026 were retrieved using the Web of Science (accessed 24 September 2026). (*) Data for 2026 are incomplete, as the year is still in progress.
